# Supplementary material for: How much we express love predicts how much we feel loved in daily life
Source: PLoS One. 2025 Jul 2;20(7):e0323326. doi: 10.1371/journal.pone.0323326 (PMC12221082; doi:10.1371/journal.pone.0323326)
Supplement: Attachment — Submitted filename: Response to Reviewers.docx [file pone.0323326.s002.docx]

Reviewers' comments:

***Reviewer #1:***

***Thank you for the opportunity to review this manuscript. As someone who researches love in romantic relationships, I was drawn to the paper from the moment I read the abstract. I looked forward to learning a bit.***

***Please note, however, my expertise does not extend to the methods employed in this study and therefore I am not able to comment on the analysis. I have informed the editor of this and suggested that they seek a second reviewer who has expertise in the methods you employ.***

***This study investigated two aspects of love (felt love and expressed love) in North American adults using EMA. If I understand correctly, the authors are particularly interested in inertia, which is a proxy for some sort of causal relationships between the two components of love, but also measures of wellbeing. Overall, I think the study is novel, interesting, and reasonably well presented. Nonetheless, there are a number of issues that I have identified with the manuscript that I think warrant either amendment or, at the very least, consideration by the authors.***

***Please note, I have included some references below. I am not suggesting that you cite these, they are just for your consideration and a starting point…***

***Intended audience
I wonder about the intended audience of the manuscript. As a romantic love researcher, I think the study is very interesting and has the potential to influence researchers’ thinking on the topic. If you are wanting to contribute meaningfully to the literature on love in romantic relationships (which you may not want to), you may think about developing your introduction and discussion. The introduction could better situate the current study within the extant literature on love (the largest area of love research is love in romantic relationships). This may involve a brief summary of the different types of love, including in romantic relationships (e.g., passionate/ romantic love and companionate love – see {Walster, 1978 #557}. This could also suggest a bit more in terms of future research. You jump into the theory of positivity resonance a bit quickly in the introduction, and I think you could situate the reader a bit better. See {Machin, 2022 #1300} or {Fehr, 1991 #1846} for the various types of love.***

Thank you for pointing this out. We are interested in contributing to love research in general, including love in romantic contexts - however it is true that we did not discuss romantic contexts in detail in the Introduction. In the revision we have elaborated more on the various contexts in which love can occur and the different types of love within each of these contexts, please see Page 3 (Introduction).

***Conceptualising love as an emotion
I also think it is narrow to describe love as a “supreme emotion.” While some researchers still apparently believe this, most contemporary researchers studying love in romantic relationships recognise love is a constellation of psychological features, including emotions, cognitions, and behaviours ({Bode, 2021 #2276} {Sternberg, 1986 #565}. I appreciate that some of the authors specialise in emotions, but this is narrowly focused and ignores the overwhelming body of evidence. Perhaps soften the language a bit to indicate that focussing on the emotional aspect of love is a valid and necessary approach. Emotion is one component of love… The authors, themselves, recognise that love is associated with “small acts or gestures.”***

This is a great point, and we appreciate the references you suggested. We have extended the Introduction to emphasize that emotion is only one aspect of love and discuss the cognitive and behavioral aspects as well, please see Pages 3-5.

***Lack of acknowledgement about the influence of different relationships
The discussion does not really detail how different types of relationships may influence the results. For example, people who live with a romantic partner or in a family situation may experience both felt and expressed love more than a single person simply because of exposure to a loved one. You may want to include acknowledgement of this, and this should be suggestive of future research. Different types of relationships may illicit different felt and expressed love, and different inertia and wellbeing.***

Thank you for this suggestion. We have added this to the Discussion and now include this line of research in our proposed future directions. Unfortunately, in the current study we did not capture what type of relationship the participants were in. However, we did capture if they were in a relationship (vs. single). Neither this relationship status nor age showed credibly non-zero correlations with any of our time dynamics parameters (see correlations and corresponding Bayes Factors in squared brackets in the table below; Bayes factors less than 1 indicate support for a correlation of zero). We added these results and data to the project OSF page. Additionally, in the revision we also acknowledge that our study does not parse out the occurrence of interactions between the participants and others (please see Discussion, Page 19).

| Time dynamics features | Relationship Status | Age |
| --- | --- | --- |
| felt love inertia | 0.118  [0.242] | -0.009  [0.173] |
| expressed love inertia | -0.068  [0.194] | 0.237  [0.698] |
| expressed to felt love effect | 0.189  [0.417] | -0.150  [0.300] |
| felt to expressed love effect | -0.031  [0.177] | -0.143  [0.284] |

***Lack of overarching theory
I think your lack of overarching theory is a major weakness of the paper.
You refer to some theories, but these are not overarching theories. They are more mechanistic (and to be honest, are not very explanatory). I refer you to the following, by some insightful researchers:***

***Many subfields within psychology lack any overarching, integrative general theoretical framework that would allow researchers to derive specific predictions from more general premises. Without a general theoretical framework, results are neither expected nor unexpected based on how they fit into the general theory and have no implications for what we expect in other domains. This situation is thrown into stark relief by comparing psychology textbooks with those in other sciences. Rather than building up principles that flow from overarching theoretical frameworks, psychology textbooks are largely a potpourri of disconnected empirical findings on topics that have been popular at some point in the discipline’s history, and clustered based on largely American and European folk categories. Outside of psychology, useful theoretical frameworks tell scientists not only what to expect, but also what not to expect (Muthukrishna & Henrich, 2019) p. 221).***

***This is the case with your manuscript. I suggest you see if the theory of evolution may be able to make better sense of your study and findings. This could be easily done by indicating what the functions of the inertia or wellbeing are in love. Why would this inertia or wellbeing have benefited reproduction or survival – once we know that, we know why it works this way. Perhaps inertia exists because love is a reciprocal process, whereby the purpose is to bond two entities together. Perhaps feeling loved is the rewarding aspect of expressed love??? Perhaps wellbeing is a way of saying that love is good for us??? I refer you to {Buss, 2019 #124}. If you don’t feel comfortable with evolution, you may be able to identify alternative theories to explain your findings. There might be a social theory that could do the job.***

This is a great point. Unfortunately, we did not make predictions based on the theory of evolution (or any other overarching theory) when designing the study. We are at this stage hesitant to “hypothesize after the results are known” (a.k.a. HARKing), but we do now reflect on how our findings align with evolutionary principles in the Discussion, Pages 17-18.

***Ideas for future research
I think you could do better with ideas for future research. This study opens up all sorts of questions. Are there sex differences. Are their age differences. Are there differences based on relationship status. Does felt or expressed love affect behavior? Many more I am sure.***

We absolutely agree that there are many more future paths of research that this study opens up. We have now explored sex, age and basic relationship status variables (please see above comment exploring relationship status). We have added more on future directions in the revised Discussion (Pages 17-19), and we are looking forward to pursuing them in the future.

***Little things
If you can, describe the participants in more detail (see {Bode, 2023 #2264}).***

We consulted this publication but unfortunately we did not collect most of the information described there. We reported sex and age in the original paper, and in the revision we added relationship status and years of education to our table with descriptives (see Page 10).

***Is there some sort of power analysis/ sensitivity analysis that can be conducted for this type of analysis? If so, add that.***

Thank you for this comment. We have updated the Procedure paragraph on Page 8 with information on power analysis.

***“Male” and “female” are biological sexes, not genders.***

Indeed, we have corrected this in the manuscript.

***Please indicate what 1 and 7 mean in the Flourishing Scale as well as 1 and 6 in the SFHS and 1 and 7 in the SHS.***

We added the words associated with the maximum and minimum numbers for the SHS and SFHS. The FS was captured on a scale from 1 to 7 and asked participants to rate their agreement with each item. This was added to the Measures section (Pages 9-10).

***Can you provide any context to the descriptive statistics. Are the means high, low, expected???***

Thank you for pointing this out. We have added more information on the descriptive statistics under the Measures section on Page 10.

***I don’t really care, but APA format says you don’t just repeat stats in text and tables…***

Thank you for pointing this out. We have removed this redundancy of restating the table values in the text.

***Figure 1 is pretty hard to make sense of. Perhaps describe it in text a bit more.***

We have clarified the description – please see updated version on Pages 13-14. We also separated the curves in the original Figure 1 into two subplots to enhance clarity.

***Your choice of the term “expressed” love is misleading/ confusing. Can you come up with a better term. Expression is suggestive of behaviour or felt love could be a form of expression.***

In the revision, we have clarified the terms and the corresponding conceptualizations to address this comment and also Reviewer 2’s similar concerns. The term “expressed love” is used as a concise abbreviation for the concept that we now more clearly articulate on Page 5.

***“These findings cast LEL as a dynamic flow of felt and expressed love which unfolds over time.” This is not a useful description. I don’t know what it means.***

Great point, we changed this sentence to:

These findings suggest that love in everyday life involves both the feelings of love and the affection we show to others, and these aspects of love continuously change and develop as time goes on.

***Unless the journal requires it, don’t have the heading Constraints of generality. See ideas for expressing issues with generalisability here: {Bode, 2023 #2264}***

We have removed this heading.

***You need a limitations paragraph, which should include generalisability issues***

We added a paragraph on limitations to the Discussion section (Page 19).

***Discuss causality in more detail (inertia and wellbeing) - what are the potential mechanisms***

We have elaborated on this in the Discussion, Page 18-19, paragraph 6.

***Reviewer #2: I appreciate having had the opportunity to review the authors’ article, entitled How much we express love predicts how much we feel loved in daily life. Because I am not an expert on the quantitative approaches utilized by the authors, I have made the theoretical contributions of the paper the primary focus of my review (with permission from the editor). Overall, I felt that the findings were very intriguing. However, I felt that the authors’ interpretations of the work required substantial revision.

• The authors consistently refer to expressed and felt love as dimensions of love experiences. In the discussion, the authors also describe love experiences in everyday life as a skill. However, although one may motivate the other, it’s not clear how both could be considered dimensions of the experience of love. As an analogy, one would not state that felt anger and behavioral aggression are dimensions of the experience of anger. Although the feeling of anger and the motivation to aggress when angry could be outputs of an anger program in the mind, it’s not clear how these are dimensions of the experience of anger; only the feeling state associated with anger (and various other, related phenomena—such as a tightening of chest, an increase in heart-rate, etc.) characterize the experience of anger. The introduction should be amended to remove this point or clarify the authors’ meaning.***

We saw expressed and felt love as integral parts of the overall experience of love and the corresponding labelling them as dimensions was motivated by the affect literature where valence and arousal are often labelled as dimensions of core affect (Russell & Barrett, 1999). Considering your arguments, we do recognize that this term is not directly applicable to love experiences, and we now refer to these as aspects.

Reference

Russell, J. A., & Barrett, L. F. (1999). Core affect, prototypical emotional episodes, and other things called emotion: Dissecting the elephant. Journal of Personality and Social Psychology, 76(5), 805–819. [https://doi.org/10.1037/0022-3514.76.5.805](https://psycnet.apa.org/doi/10.1037/0022-3514.76.5.805)

***• The authors write that “on average, felt love decayed at a slower rate than the expressed love, suggesting a stronger lingering effect of felt love than expressing love” and discuss this in the General Discussion. However, in addition to not representing two dimensions of love experiences, it's not clear that these phenomena are directly comparable in the first place. Felt love is an affective state, whereas expressed love is an action. Comparing the decay of one to the decay of the other is analogous to comparing decays in acts of aggression to decays in internal feelings of anger; they’re completely different phenomena. Thus, it’s not clear how one could interpret this comparison.

One alternative possibility is to compare felt love and expressed love to findings from other published research. For instance, if felt love/expressed love decayed at a slower or faster rate than has been suggested in previous research on felt hatred/expressed hatred, this could be very interesting. If no such comparisons are available in the literature (or if such comparisons are inappropriate in the opinion of the authors), this is not needed. However, in either case, any comparisons between felt and expressed love should be removed or explicitly justified in the manuscript.***

Thank you for this great point. To address your comment, first we have removed the original direct comparisons between felt and expressed love inertia values. We revised the corresponding part in the Discussion section and we focus on the magnitude of these inertia values. Moreover, we added comparisons to other constructs, namely affect dynamics. Please see the revised parts on Pages 16-17.

***• The authors mention projection as a potential mechanism for the relationship between felt and experienced love, but other interpretations are possible. In particular, expressions of love could lead to others expressing more love in return, and this reciprocity could increase felt love. This possibility should be discussed by the authors.***

This is a great point. We have added this possibility to the Discussion:

Importantly, the expressed to felt love cross-influence provided evidence of love dynamics influencing one another. This cross-influence indicated expressed love could be a potential route to increase felt love. One possible explanation for this finding is that by expressing more love, an individual may also receive more expressions of love in return, resulting in more felt love.

***• The limitations section of the General Discussion is extremely short and omits a number of key issues, such as the small sample size, ambiguities in the interpretation (described above), the use of single-item measures, the absence of an item measuring others’ expressions of love toward the participant (e.g., one could feel unloved despite others attempting to express love—just as one could feel lonely despite interacting with others), and so on. These limitations should be addressed in the limitations section.***

Thank you for pointing this out. We are now discussing these issues in a new section labelled Limitations (Page 19).

***Other points:
• Specify how many days the study lasted.***

Great point. We have changed “four weeks” in the text to 28 days.

***• Indicate when each question was answered. For instance, was the subjective happiness scale completed only in the first session, only in the last session, during a random session, or multiple times?***

For the trait-level scales, the participants answered these questions before the EMA part of the study during an intake session. The two love items were asked multiple times a day. Thank you for pointing out that this wasn’t clearly stated. We have amended the Procedures section on Pages 8-9 to clarify this.

• ***Specify the meaning of the 0-100 scales used. For instance, when asking “Since the last survey, I have been expressing love,” what were the labels on the endpoints? What were participants told in terms of what a score of 0 or 100 should be taken to mean?***

We have now added this information under Measures on Page 9: Individuals responded on a sliding scale from 0 to 100, and a label of “Not at all” was shown above the left side of the scale, corresponding to 0, while “Extremely” was displayed above the right side of the scale, corresponding to 100.

***• In the first sentence, “has been elevated to the status of a ‘Supreme emotion’” should say “has been elevated by some researchers…” It's not clear what it means to refer to one emotion as "Supreme" relative to others, and this position is not universal in the field.***

Thank you for pointing this out. This was a wording we took from the referenced literature. To avoid confusion, we now explain that the authors meant that love is a unique emotion in that it can impact human survival through building deep connections and relationships, surpassing other emotions (Fredrickson, 2016).

We have updated the text when introducing love as a “supreme emotion”:

For instance, love has been studied as a temporally dynamic burst of emotion shared between individuals, leading some researchers to regard love as a “supreme emotion” (Fredrickson, 2016; Zhou et al., 2022).

***• Reword this sentence to be grammatically correct (change vs. changes): “Higher inertia means that the level of loving feelings change more slowly, while lower inertia allows for quick regulation back to baseline.” Additionally, change the word “allows” to “refers to”.***

Thank you for pointing this out. We have corrected this sentence.

***• Add a “the” in the following sentence: “Conceptually, this means that more intensely participants expressed love, the stronger they were feeling loved, with this dynamic peaking around three hours.”***

We have corrected the above sentence. Thank you for pointing this out.

***Reviewer #3: This is an interesting and important paper that illuminates new patterns for and between expressing love to another and feeling loved by another. The analyses appear to be well justified and described and the interpretations of them are accessibly communicated.***

***One area of murkiness that I would strongly advice be clarified is the term "felt love" which is ambiguous as to whether it reflect the love one person feel FROM another or FOR another. I understand that the author intend this variable to reflect the former (FROM another) yet in affective science, it is routine to describe a single emotional state in terms of the degrees to which it is FELT and/or EXPRESSED. Readers steeped in that tradition are likely to interpret "felt love" as love felt (but not necessarily expressed) FOR another.***

This is a great point, thank you! We clarify this issue now when we introduce the conceptualization on Page 5. It now reads as:

Consistent with research on love and loving-kindness (e.g. Condon & Makransky, 2020; Kahana et al., 2021), we distinguish between two aspects of LEL: a felt aspect, that captures how much a person feels loved when receiving love from someone, and an extending aspect, that focuses on the behavior of a person expressing loving feelings towards someone. We label the felt aspect of love as “feeling loved” or “felt love,” and the extending aspect of love as “expressing love” or “expressed love.”
